# Clinicians’ beliefs and attitudes toward patient self-management in the Netherlands; translation and testing of the American Clinician Support for Patient Activation Measure (CS-PAM)

**DOI:** 10.1186/s12913-015-0799-y

**Published:** 2015-04-03

**Authors:** Jany Rademakers, Daphne Jansen, Lucas van der Hoek, Monique Heijmans

**Affiliations:** NIVEL – Netherlands Institute for Health Services Research, PO Box 1568, 3500 BN Utrecht, The Netherlands

**Keywords:** Patient activation, Self-management, Health care provider

## Abstract

**Background:**

The aim of this study was to test the Dutch version of the Clinician Support for Patient Activation Measure (CS-PAM), to explore the beliefs of Dutch clinicians about patients’ self-management, and to establish whether there are differences in this respect between general practitioners and other primary care providers.

**Methods:**

The CS-PAM was translated in Dutch and data were collected in a sample of 489 general practitioners and other primary care providers. Statistical analyses (RASCH, Cronbach’s α) were performed to establish the psychometric properties of the instrument.

**Results:**

The psychometric scores of the Dutch CS-PAM were acceptable to good, and the difficulty level and structure was comparable to that of the original instrument. The average score of Dutch clinicians on the CS-PAM was 65.1 (SD 10.7), somewhat lower compared to their colleagues in the US (69; SD 12.1) and the UK (69, SD 12.8). Dutch general practitioners scored significantly lower on the CS-PAM compared to other primary care providers.

**Conclusions:**

The Dutch CS-PAM is a reliable instrument to measure beliefs of clinicians regarding patient self-management. Further validation studies are necessary to establish the distribution of scores in specific provider populations and to assess the clinical relevance of the instrument for different outcomes.

**Electronic supplementary material:**

The online version of this article (doi:10.1186/s12913-015-0799-y) contains supplementary material, which is available to authorized users.

## Background

Chronic illnesses (such as cardiovascular diseases, chronic respiratory diseases and diabetes) are the main cause of death worldwide (63%) and prevalence rates are rising [1]. The growing number of chronic patients weighs heavily on healthcare systems, both with respect to the necessary capacity of care providers as to increasing costs [2,3]. Self-management is seen as one of the major solutions for these healthcare problems. Though the definitions of self-management vary, central is the active engagement of the individual patient (and his or her family) to participate in the care for their own illness. It involves medical aspects, such as taking medication or handling symptoms, but also coping with the illness and related problems in daily life. Self-management has indeed been linked to less use of health care services and lower costs [4-6]. On the individual level patient self-management has proved to have positive effects on health outcomes such as quality of life, compliance and lifestyle [5,7-9]. Furthermore, sharing responsibilities between patient and provider may improve patient involvement in and a more patient-centered organization of healthcare delivery. Therefore in the Netherlands, as in many other western countries, there is a growing consensus that chronic patients should be active partners in the management of their own health and healthcare. Self-management of chronic patients is strongly recommended in care standards and through the promotion of individualized care plans.

However, despite the encouraging evidence and although the idea of self-management receives support from many stakeholders, not all patients and their caregivers are embracing the concept. Especially older people and patients with lower health literacy skills or lower activation levels are known to be less effective self-managers [10-13]. These groups have more and specific self-management support needs, which are often not met by their care providers [14,15]. As in other western countries such as the US and the UK [16], many clinicians in the Netherlands have not yet adjusted to the new role of the patient as active partner in the care process, which requires a more coaching attitude and supportive (instead of directive) behavior from them. They are hesitant to encourage patients’ self-management: individualized care plans are hardly used and many clinicians regard self-management support as too time consuming or not suitable for an important part of their patients [17,18].

In order to better understand why implementation of self-management and self-management support is such a difficult process it is important to improve our insight into the patients’ and clinicians beliefs and attitudes about self-management. Recently we studied Dutch patients’ beliefs about self-management and active involvement in their own care. In order to do so, we translated and validated the Patient Activation Measure (PAM) [19,20]. The PAM is a 13-item instrument which assesses a patient’s self-reported knowledge, skills and confidence for self-management of one’s health or chronic condition, developed by Hibbard et al. in the United States [21,22]. The PAM divides patients into one of four progressively higher activation levels, which are associated with specific self-management and other health related behaviors. In a sample of Dutch healthcare consumers, 22.0% scored within the first (and lowest) activation level and 25.9% in level 2, indicating that one-fourth to half of the general population experiences difficulties with self-management [20]. In the validation studies in the Netherlands, higher PAM scores were associated with more health information use [20], more active provider choice [23], better participation of chronic patients in medical consultations [24], and better self-management in diabetes patients [25]. These results confirm earlier US studies with the instrument.

**Figure 1:**
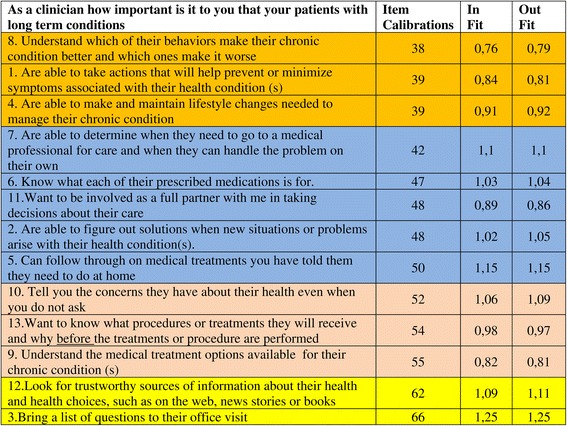
**Item calibrations, in-fit and out-fit per item of the Dutch CS-PAM.**

Since self-management of patients requires clinician support, it is also essential to know how general practitioners and other primary care providers actually think about a more active role for their patients. In the Netherlands there was no reliable instrument to measure the beliefs of Dutch clinicians about patients’ self-management, therefore we decided to translate and validate the Clinician Support for Patient Activation Measure (CS-PAM) for use in the Netherlands as well. The CS-PAM is adapted from the PAM and assesses a clinician’s beliefs and attitudes about the importance of patient self-management [16]. The PAM focuses on the various patient competencies needed to successfully manage one’s health or chronic illness. The items of the CS-PAM explore the degree to which clinicians regard the same patient competencies as important. The specific content of the items is described in Figure 1. In the original development study in a sample from both the US and the UK, the CS-PAM proved to be a reliable instrument which is able to assess and differentiate between clinicians in the level to which they endorse patient self-management [16]. The term clinician in this context can refer to several types of professional caregivers. In the Netherlands, most chronic care is provided in general practice. Since patients in that setting above all have contact with practice assistants and specialized practice nurses for the monitoring and treatment of their chronic condition, we hypothesized these care providers to have more affinity with the concept of patient self-management compared to general practitioners. Therefore we examined possible differences between general practitioners and other primary care providers (practice assistants and nurses) with respect to their beliefs and attitudes regarding self-management.

Our study goals thus were:

- to establish the psychometric properties of the Dutch version of the CS-PAM;
- to gain insight into which elements of a patient’s role in self-management are most strongly and least strongly endorsed by clinicians in general practice in the Netherlands and
- whether the level of endorsement differs by the specific function of the clinician.

## Methods

### Translation and adaptation process

The CS-PAM was translated and adapted using a systematic approach conforming WHO instructions, which was also used for the translation of the PAM [19,26]. This method includes the following steps: forward translation by two independent Dutch translators, expert panel meeting, backward translation by a different translator who does not know the original instrument, and consensus about the final version. The expert panel included both translators and three researchers with an expertise in chronic care, patient activation and measurement development. Discrepancies between the two translations were discussed and resolved. The concept instrument was then translated back into English. There were few minor discrepancies between the backward translation and the original instrument, which lead to the approval of the Dutch translation. The final version of the instrument (CS-PAM Dutch) is attached as an Additional file to this paper (Additional file 1; copyright Insignia Health; www.InsigniaHealth.com).

For our study we used a 13-item version of the CS-PAM, which was provided to us by Insignia Health (see Acknowledgements). Two elements were different in the instrument we tested from the CS-PAM version originally described by Hibbard et al. [16]. Item 6 of the earlier version (‘Believe when all is said and done that they are the ones who are responsible for managing their health’) had been omitted from the questionnaire by the developers. Also the order of the items in the current CS-PAM questionnaire was different from the one described in Hibbard et al. [16]. The order in that publication was established by performing RASCH-analyses (see analyses section) and progressively describes items that are most endorsed to least endorsed by UK and US clinicians. In Figure 1, the 13-item version of the CS-PAM and the numbers of the items (1-13; reflecting the order in which they were presented in our study) are shown.

### Questionnaire

Following the translation procedure, the final version of the Dutch CS-PAM (see Additional file 1) was incorporated in a larger questionnaire on self-management and self-management support and send by mail to three samples of primary practices (see Participants).

### Participants

In this study, three types of clinicians were included: doctor’s or practice assistants (who predominantly perform administrative tasks in general practices but also support patients e.g. with respect to information needs), practice or specialized nurses (who work in general practices and have specialized in the care for a chronic disease e.g. asthma/COPD or diabetes) and general practitioners. For the recruitment of the clinicians in this study we used three different sources:

1. All general practices who had contributed to the selection of chronic disease patients for the Dutch National Panel of people with Chronic illness or Disability (NPCD) between 2009 and 2013 were approached to participate in this study. The NPCD is a nationwide prospective panel-study in the Netherlands. NPCD consists of over 2.500 people aged 15 years and over with medically diagnosed chronic disease(s) and/or moderate to severe levels of physical disability. It has been set up to provide information with respect to the consequences of chronic illness and disability from the patient’s perspective. A total of 112 practices received the questionnaire of which 65 practices responded (58%).
2. A random sample of 500 general practices was drawn from the NIVEL National Registration of General Practitioners. In this registration -which originates from 1974- all Dutch general practices are included. Of these 496 practices, 147 practices returned the questionnaire (30%).
3. A study focusing on diabetes care in which four diabetes care groups participated. A care group entails multiple primary care practises. In these care groups, 434 professionals were directly invited to participate in the study. In two care groups the general practitioners were asked to further distribute the questionnaire to another professional in their practice. It is unknown how many care providers were accessed in this way.

From each practice in each of the subsamples, multiple clinicians could participate in the study.

The study does not fall within the scope of the Dutch Medical Research Involving Human Subjects Act and therefore does not require ethical approval.

### Statistical analyses

The psychometric properties of the Dutch CS-PAM were analyzed using the RASCH-model, which is a psychometric model for analyzing categorical data, such as answers to questions on an assessment or questionnaire responses [27]. Through RASCH analyses the difficulty level of individual items and their order can be established and interval-level, unidimensional, probabilistic Guttman like scales can be created. The calibration of an item indicates how difficult it is for respondents to endorse it: with respect to the CS-PAM, it means that the higher the calibration score the more difficult for the clinician to agree to that specific item. The item fit statistics (in-fit and out-fit) describe how accurately or predictably the responses to that item fit the model. In-fit relates to the inlier sensitivity of the item (for items with a difficulty close to the person), out-fit to the outlier sensitivity (for items with a difficulty far from the person). A fit value of 1,0 indicates a perfect fit, and fit values between 0,5 and 1,5 are considered to be acceptable. The personal reliability is the measure which describes the degree to which an individual’s response pattern conforms to the model.

To determine the internal consistency of the instrument, Cronbach’s α was used. Differences between the scores of general practitioners and other care providers were established with a regression analysis.

## Results

In total 496 clinicians returned a questionnaire (Table 1).

**Table 1:** **Distribution of types of clinicans over the study samples (N and %)**

	**NPCD**	**GP national registration**	**Diabetes study**
General practitioner	74 (67%)	144 (59%)	80 (57%)
Practice assistant	17 (15%)	52 (21%)	29 (20%)
Practice nurse	13 (12%)	31 (13%)	28 (20%)
Specialized nurse	5 (4%)	4 (2%)	3 (2%)
Doctor’s assistant	2 (2%)	13(5%)	1 (1%)
**Total**	**111 (100%)**	**244 (100%)**	**141 (100%)**

Seven questionnaires were omitted from analyses because the scores on all items were missing. Therefore the N in the study was 489. The total sample of clinicians (60.8% female) had on average 13.9 years of experience. GP’s worked on average 17.8 years in this position (the information on experience was not available for the participants from the Diabetes study; there were no differences between the other subsamples). The three subsamples did not differ with respect to background characteristics such as age and gender.

Cronbachs α of the CS-PAM was computed for the three different study samples and was .97 (NPCD), .82 (GP national registration) and .83 (Diabetes study), which indicates a good to very good internal consistency of the instrument. In Additional file 2, the descriptive statistics on each of the items (means, standard deviations and number of observations) are presented.

In Figure 1, the results of the RASCH analysis are presented. It provides an overview of the items clinicians find easier or harder to agree with.

The item-calibrations in our study are distributed between 38 and 66, on a theoretical 0-100 scale indicating the level to which it is easy (0) or difficult (100) for clinicians to agree with that specific item.

**Figure 2:**
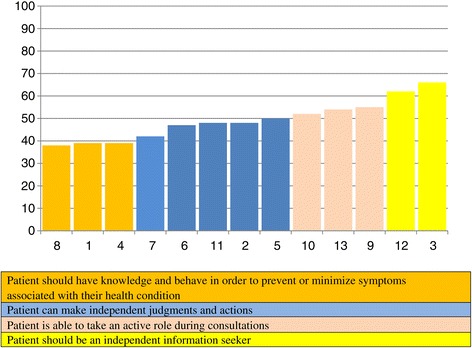
**Difficulty structure of the Dutch CS-PAM.**

On the basis of the difficulty structure of the Dutch CS-PAM, the items can be categorized in four groups (Figure 2). Group 1 are the relatively ‘easy’ items with low calibrations (8, 1, 4), whereas group 4 are the most ‘difficult’ items with the highest item calibrations (12, 3).

Though the order of the variables within the groups is slightly different in the Dutch sample most items fall within the limits of the original four item groups described by Hibbard et al. [16]. Only two items are located on a really different position (defined as > 5 points difference in calibration scores): item 7 (Are able to determine when they need to go to a medical professional for care and when they can handle the problem on their own) is more endorsed by Dutch clinicians compared to their US/UK colleagues, while item 5 (Can follow through on medical treatments you told them they need to do at home) is much less endorsed in the Netherlands. Partly as a consequence of this, the original group labels did not fit and were –on the basis of their content- adjusted to the Dutch situation. The in-fit and out-fit measures of the items were all between .76 and 1.25, which is within acceptable limits (0.5 and 1.5). This indicates that there are few unexpected patterns of observations in the sample. The person reliability was .82.

The average score on the Dutch CS-PAM for the total group of clinicians was 65.1 (SD 10.7). This is somewhat lower compared to the US (69; SD 12.1) and UK sample (69, SD 12.8) [16]. In Table 2, the average score on the Dutch CS-PAM is shown for general practitioners and other primary care providers. Because of the small numbers, we have taken the ‘other primary care providers’ together in the comparison.

**Table 2:** **Average score on the CS-PAM (+ SD) per subsample and type of provider**

	**NPCD**	**GP national registration**	**Diabetes study**	**Total**
General practitioner	61.2 (9.2)	63.7 (10.1)	65.8 (12.1)	63.7 (10.6)
Other primary care providers	67.6 (11.7)	65.5 (9.1)	69.7 (11.7)	67.2 (10.5)
**Total**	**63.3 (10.5)**	**64.4 (9.7)**	**67.5 (12.0)**	**65.1 (10.7)**

In the total sample, general practitioners scored significantly lower on the Dutch CS-PAM compared to other primary care providers (p < .000) (Table 2).

## Discussion

The results of our study demonstrate that the Dutch CS-PAM is a reliable instrument to measure clinicians’ beliefs and attitudes towards patient self-management. Psychometric scores (internal consistency, personal reliability and fit values) are acceptable to good. The item calibration scores are similar to that of the development study [16], which means that the difficulty level of the items in the CS-PAM (i.e. the likelihood that they could endorse them) is comparable for the clinicians in the three countries that were included in these two studies (US, UK and the Netherlands). The average score of the Dutch clinicians on the CS-PAM, however, is somewhat lower compared to their US/UK colleagues.

On the basis of the difficulty structure of Dutch CS-PAM also four groups of items could be established. However, two items had really different positions compared to the development study. Dutch clinicians find it easier to endorse (and thus more important) that patients with long term conditions are able to determine when they need to go to a medical professional for care and when they can handle the problem on their own, compared to their US and UK colleagues. On the other hand, they are less likely to endorse the statement that it is important that patients can follow through on medical treatments they told them to do at home. Both response patterns might signify a slightly different position and attitude of the Dutch clinician in general practice. Since he (or she) is not paid per visit (as is the case in the US) there is no reason that patients should visit the practice for problems they could also handle themselves. It would only add to the clinician’s already heavy workload. The fact that they are less likely to endorse the statement on following through on medical treatments might have to do with the ‘paternalistic’ tone of this item. The Dutch translation might emphasize this sentiment, compared to the original version. Dutch general practitioners (and especially practice assistants and nurses) might resent this tone and will therefore be less willing to agree with it.

Because of the different order of some items, specifically the fact that following through on medical treatments is much less agreed with as important by Dutch clinicians, we have relabelled item group 1: ‘Patient should have knowledge and behave in order to prevent or minimize symptoms associated with their health condition’ (in the development study it was ‘Patients should follow medical advice’). Furthermore, because all three items of group 3 pertain to the patient’s role during the patient-care provider interaction, we consider being ‘able to function as a member of the care team’ (the original label of this group) as too general and have relabelled it: ‘Patient is able to take an active role during consultations’.

Looking at the content of the items, it is clear that items focusing on knowledge and basic self-management skills (including lifestyle adjustments) are the easiest to endorse for Dutch clinicians and thus considered the most important aspects of self-management, while items requiring a greater level of independent judgement or action are less likely agreed to. Items which focus on an active role of patients in consultations are even less endorsed. And items that pertain to the patient as an active and independent information seeker are most difficult to agree to and therefore considered least important by most Dutch clinicians. This hierarchy in their beliefs and attitudes regarding patient self-management is similar to US and UK clinicians.

Compared to other primary care providers such as practice assistants and specialized nurses, general practitioners in the Netherlands on average score lower on the CS-PAM. It is important to further study the causes of these differences, e.g. whether they are influenced by the clinician’s appraisal of their own skills and opportunities to support self-management. In that case, the beliefs and attitudes of general practitioners could be negatively influenced by the lack of time they experience during a consultation. Also the training of other primary care providers might have been more targeted at communication and coaching skills giving them the means to put their beliefs into practice, whereas especially in more traditional medical schools this type of education would be lacking.

This study has shown that the Dutch CS-PAM is a reliable instrument to measure beliefs and attitudes of clinicians regarding patient self-management. However, it does not give us insight in the distribution of scores in a representative sample of clinicians. The response in the representative subsample was low (30%) and the subsamples of NPCD and the Diabetes study might be biased towards more positive beliefs since these clinicians are extra involved in the recruitment or treatment of people with chronic illness. Further validation studies are necessary to establish the distribution of scores in specific provider populations and to assess the clinical relevance of the instrument for different outcomes. Amongst others, one of these outcomes should be the actual level of support and coaching the clinicians provide their patients, thus establishing the predictive validity of the instrument. After proper validation, the Dutch CS-PAM will be a useful research instrument to assess the level of concordance between current policy in the Netherlands which is focused on a more active role for the patient and the beliefs of (specific groups of) clinicians which may be more hesitant in this respect. The CS-PAM could further be used in the development and evaluation of interventions for clinicians that focus on more adequate self-management support and better coaching skills, both as a screener and as an outcome measure.

## Conclusions

The Dutch CS-PAM is a reliable instrument to measure beliefs of clinicians regarding patient self-management. Further validation studies are necessary to establish the distribution of scores in specific provider populations and to assess the clinical relevance of the instrument for different outcomes.

## Additional files

Additional file 1:
**CS-PAM (Dutch version).**
Additional file 2:
**Descriptive statistics (mean, SD and number of observations) per item of the Dutch CS-PAM.**
