# Supplementary material for: Clinicians’ beliefs and attitudes toward patient self-management in the Netherlands; translation and testing of the American Clinician Support for Patient Activation Measure (CS-PAM)
Source: BMC Health Serv Res. 2015 Apr 3;15:138. doi: 10.1186/s12913-015-0799-y (PMC4419501; doi:10.1186/s12913-015-0799-y)
Supplement: Additional file 1: — CS-PAM (Dutch version). [file 12913_2015_799_MOESM1_ESM.docx]

Zorgverleners hebben verschillende opvattingen en verwachtingen over hun patiënten. Beantwoord de onderstaande uitspraken voor zover ze van toepassing zijn op u en uw praktijk. Als de uitspraak niet van toepassing is, kies dan n.v.t.

Hoe belangrijk is het voor u als zorgverlener dat uw patiënten met chronische aandoeningen:

| 1. In staat zijn om maatregelen te nemen die symptomen van hun aandoening(en) kunnen voorkomen of beperken. | Niet belangrijk | Enigszins belangrijk | Belangrijk | Uitermate belangrijk | n.v.t. |
| --- | --- | --- | --- | --- | --- |
| 2. In staat zijn om oplossingen te bedenken als zich bij hun aandoening(en) nieuwe situaties of problemen voordoen. | Niet belangrijk | Enigszins belangrijk | Belangrijk | Uitermate belangrijk | n.v.t. |
| 3. Een lijst met vragen meebrengen bij een bezoek aan de praktijk. | Niet belangrijk | Enigszins belangrijk | Belangrijk | Uitermate belangrijk | n.v.t. |
| 4. In staat zijn om aanpassingen in hun leefstijl te maken en vol te houden die nodig zijn om met hun chronische aandoening om te gaan. | Niet belangrijk | Enigszins belangrijk | Belangrijk | Uitermate belangrijk | n.v.t. |
| 5. Medische behandelingen kunnen uitvoeren waarvan u gezegd heeft dat ze ze thuis moeten doen. | Niet belangrijk | Enigszins belangrijk | Belangrijk | Uitermate belangrijk | n.v.t. |
| 6. Weten waar elk van hun voorgeschreven medicijnen voor dient. | Niet belangrijk | Enigszins belangrijk | Belangrijk | Uitermate belangrijk | n.v.t. |
| 7. In staat zijn om te beoordelen wanneer ze professionele zorg moeten inschakelen en wanneer ze een probleem zelf kunnen aanpakken. | Niet belangrijk | Enigszins belangrijk | Belangrijk | Uitermate belangrijk | n.v.t. |
| 8. Begrijpen welk gedrag hun chronische aandoening verbetert of juist verergert. | Niet belangrijk | Enigszins belangrijk | Belangrijk | Uitermate belangrijk | n.v.t. |
| 9. Begrijpen welke verschillende medische behandelmogelijkheden beschikbaar zijn voor hun chronische aandoening(en). | Niet belangrijk | Enigszins belangrijk | Belangrijk | Uitermate belangrijk | n.v.t. |
| 10. Hun zorgen over hun gezondheid aan u vertellen, zelfs als u daar niet om vraagt. | Niet belangrijk | Enigszins belangrijk | Belangrijk | Uitermate belangrijk | n.v.t. |
| 11. Als volwaardige partner betrokken willen zijn bij het nemen van beslissingen over hun zorg. | Niet belangrijk | Enigszins belangrijk | Belangrijk | Uitermate belangrijk | n.v.t. |
| 12. Zoeken naar betrouwbare informatiebronnen over hun gezondheid en gezondheidskeuzes, bijvoorbeeld op internet, in nieuwsberichten of in boeken. | Niet belangrijk | Enigszins belangrijk | Belangrijk | Uitermate belangrijk | n.v.t. |
| 13. Willen weten welke procedures of behandelingen ze gaan krijgen en waarom, vóórdat deze worden uitgevoerd. | Niet belangrijk | Enigszins belangrijk | Belangrijk | Uitermate belangrijk | n.v.t. |
