# Supplementary material for: Clinicians’ beliefs and attitudes toward patient self-management in the Netherlands; translation and testing of the American Clinician Support for Patient Activation Measure (CS-PAM)
Source: BMC Health Serv Res. 2015 Apr 3;15:138. doi: 10.1186/s12913-015-0799-y (PMC4419501; doi:10.1186/s12913-015-0799-y)
Supplement: Additional file 2: — Descriptive statistics (mean, SD and number of observations) per item of the Dutch CS-PAM. [file 12913_2015_799_MOESM2_ESM.docx]

Appendix B

Descriptive statistics (mean, SD and number of observations) per item of the Dutch CS-PAM

| **As a clinician how important is it to you that your patients with long term conditions** | **Mean score** | **SD** | **n** |
| --- | --- | --- | --- |
| 1. Are able to take actions that will help prevent or minimize symptoms associated with their health condition (s) | 3.6 | .52 | 488 |
| 2. Are able to figure out solutions when new situations or problems arise with their health condition(s). | 3.2 | .65 | 489 |
| 3.Bring a list of questions to their office visit | 3.5 | .76 | 487 |
| 4. Are able to make and maintain lifestyle changes needed to manage their chronic condition | 3.6 | .55 | 487 |
| 5. Can follow through on medical treatments you have told them they need to do at home | 3.2 | .66 | 486 |
| 6. Know what each of their prescribed medications is for. | 3.3 | .64 | 489 |
| 7. Are able to determine when they need to go to a medical professional for care and when they can handle the problem on their own | 3.5 | .59 | 489 |
| 8. Understand which of their behaviors make their chronic condition better and which ones make it worse | 3.6 | .51 | 488 |
| 9. Understand the medical treatment options available for their chronic condition (s) | 3.0 | .65 | 487 |
| 10. Tell you the concerns they have about their health even when you do not ask | 3.1 | .67 | 488 |
| 11.Want to be involved as a full partner with me in taking decisions about their care | 3.3 | .65 | 485 |
| 12.Look for trustworthy sources of information about their health and health choices, such as on the web, news stories or books | 2.7 | .74 | 488 |
| 13.Want to know what procedures or treatments they will receive and why before the treatments or procedure are performed | 3.0 | .70 | 486 |
